# Closed Irrigation in Pyogenic Flexor Tenosynovitis: A Practical Angiocatheter Modification and Review of Irrigation Aids

**DOI:** 10.7759/cureus.100453

**Published:** 2025-12-30

**Authors:** Ioannis Sarris, Konstantinos Papadopoulos, Dimitrios Georgou, Kyriakos Papavasiliou, Eleftherios Tsiridis

**Affiliations:** 1 3rd Academic Orthopaedic Department, Papageorgiou General Hospital of Thessaloniki, Aristotle University of Thessaloniki, Thessaloniki, GRC; 2 Center of Orthopaedic and Regenerative Medicine - Center of Interdisciplinary Research and Innovation, Aristotle University of Thessaloniki, Thessaloniki, GRC

**Keywords:** angiocatheter modification, closed irrigation, hand infection, pyogenic flexor tenosynovitis, tenosynovitis/surgery

## Abstract

Pyogenic flexor tenosynovitis is a severe infection of the upper limb. While early mild cases may be treated conservatively, surgical intervention remains the standard of care for more advanced presentations. Closed irrigation techniques, typically performed using angiocatheters, are increasingly preferred over open approaches.

We present a simple and widely accessible modification of an angiocatheter intended to facilitate multidirectional irrigation during closed catheter washout. This report does not evaluate irrigation efficiency, and any potential advantages remain theoretical. In addition, we compile available references on instruments and modifications intended to improve irrigation efficiency.

This modification is feasible but unvalidated, and any theoretical benefits regarding fluid distribution or irrigation efficiency remain speculative. Furthermore, this report consolidated multiple previously described modifications into a single reference source.

Our modification is low-cost, reproducible, and easily performed using commonly available tools. While it may offer a practical option during surgery, its clinical, mechanical, and fluid-dynamic performance has not been evaluated. Further research is required to assess safety, efficacy, and optimal design parameters.

## Introduction

Pyogenic flexor tenosynovitis (PFT) is a bacterial infection of the flexor tendon sheath. The closed tendon sheath creates a confined environment in which infection can spread quickly along the digit. Rapid progression of inflammation, purulence, tendon ischemia, and permanent functional loss if not promptly treated is not uncommon [1].

Despite its severity and its reported incidence of up to 10% of all hand infections, a lack of consensus regarding the optimal management protocol and the timing of interventions remains [2]. Although antibiotics and other conservative measures are increasingly advocated as first-line treatment, surgical debridement and irrigation are still widely considered the cornerstone of management in acute PFT [1].

There are two main entities in the surgical approach to debridement and irrigation: the open technique and the closed technique, both of which have been described in detail [3]. Closed irrigation is widely used but remains technically challenging in certain cases. Irrigation is conducted through two small incisions: one vertical incision at the A1 pulley level and a semi-Brunner incision at the A5 pulley level [4]. A number of tools are used, with the main instrument being the angiocatheter. Irrigation reduces the number of microorganisms in the closed space of the tendon sheath and disrupts any purulent septations, thereby preventing the spread of infection in radial and ulnar bursae or even to Parona’s space [5].

A simple, cost-effective, and widely available modification of angiocatheters used for irrigation is presented by our team to achieve potentially more effective irrigation in cases of PFT through multidirectional outflow patterns, without the need for specialized tools that are not widely available in the operating rooms. The modification has not undergone clinical or laboratory validation, and any perceived advantages remain hypothetical.

Moreover, we have sought to summarize in a single report the available references regarding instruments or modifications designed to facilitate more efficient irrigation.

## Technical report

**Figure 1:**
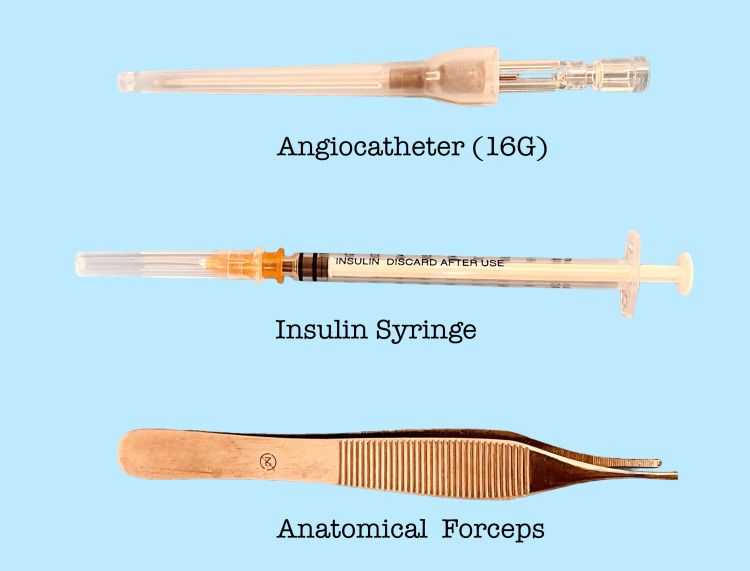
Instruments needed for adjuvant preparation.

Modification of the angiocatheter is performed aseptically before the initial incisions. A 16G (1.7 mm × 30 mm) angiocatheter is used; this selection reflects routine availability, operator familiarity, and a balance between stiffness for insertion and adequate lumen diameter for irrigation rather than evidence-based optimization. For the modification, anatomic forceps and a 0.3 mL (30 G × 8 mm) insulin syringe are required (Figure 1).

**Figure 2:**
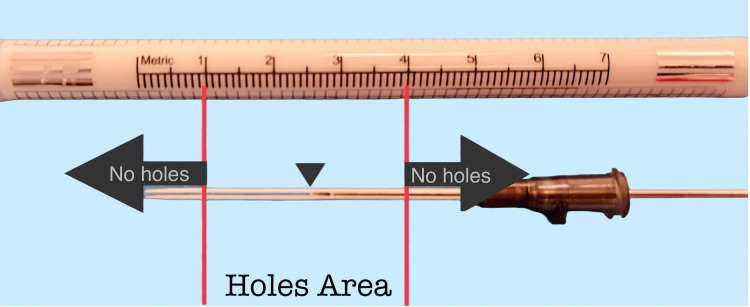
No-hole areas (arrows). Catheter tip (arrowhead).

We partially retract the guide-wire to the midpoint of the tube and hold the free tip of the tube with anatomic forceps. No holes are created near the catheter tips to preserve its stiffness. Hole creation with the insulin needle begins approximately 10 mm away from the catheter stem and ends 10 mm before the catheter tip (Figure 2).

**Figure 3:**
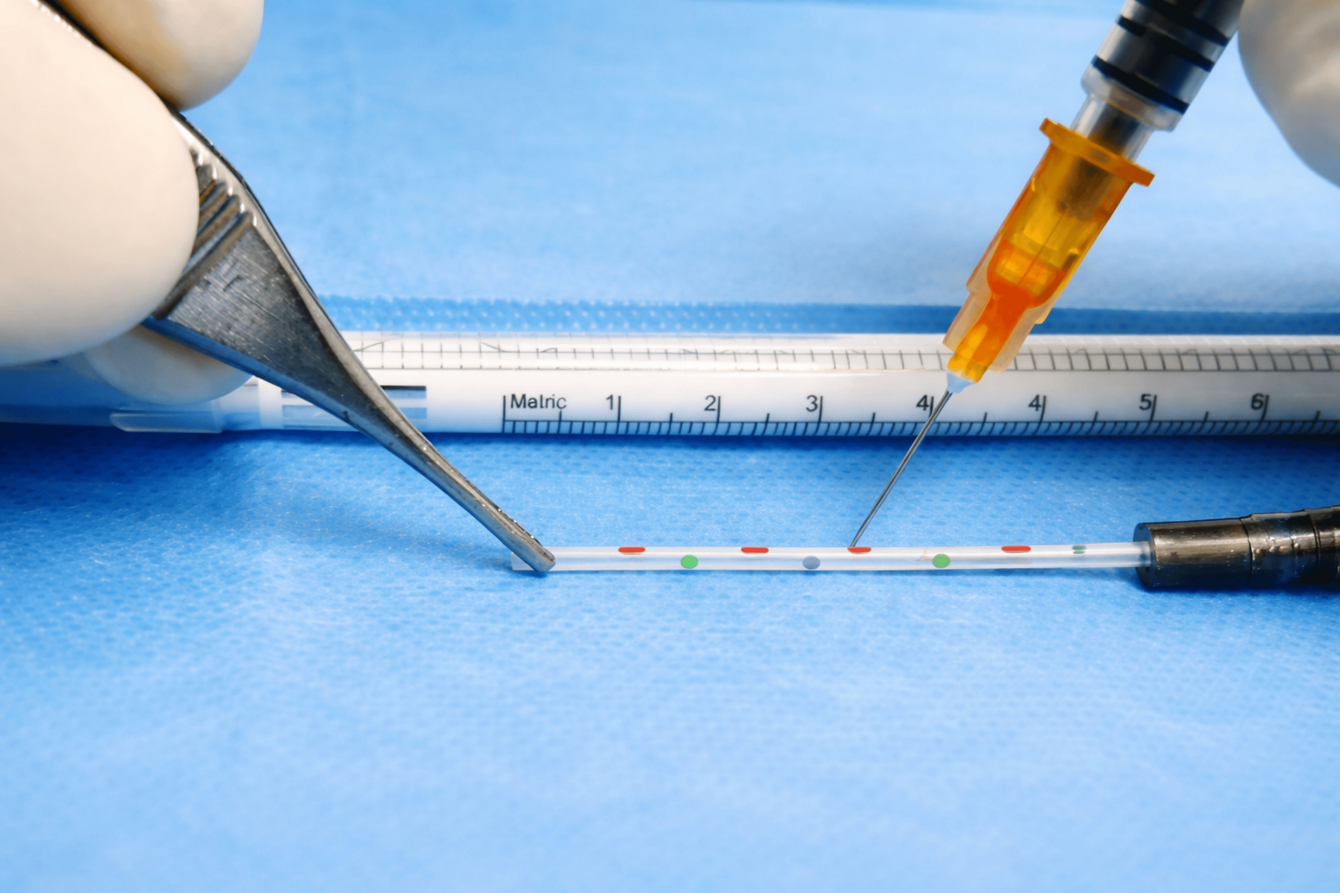
Multidirectional hole creation along the catheter segment. Using a 30 G insulin needle, holes are drilled at approximately 5 mm intervals in multiple directions to enhance outflow. Holes are created while gradually retracting the guide wire to avoid needle interference and preserve catheter integrity. All modifications are performed within the sterile field using sterile instruments. Different hole colors indicate different hole directions.

Holes are drilled in multiple directions to achieve wider outflow, progressing from the catheter periphery toward the center while gradually retracting the guide-wire, in order not to interfere with the insulin needle. The distance between holes is proposed to be roughly 5 mm using a 30 G insulin needle. This spacing is based on practical ease of placement and empirical testing in order not to compromise the catheter’s structural integrity. All modifications are performed aseptically within the sterile field using sterile instruments (Figure 3).

**Figure 4:**
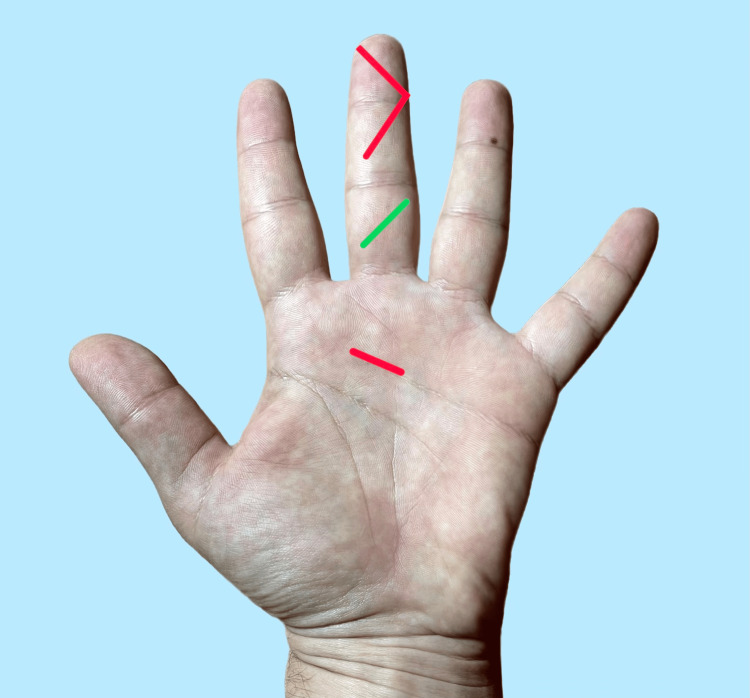
Proposed incisions for closed irrigation (red). Additional subcutaneous incision (green). Original image created by the authors for this study.

After preparation of the catheter and application of a pneumatic tourniquet, the operative field is prepared and draped in a sterile fashion. Two incisions are made: a vertical incision over the A1 pulley and a semi-Brunner incision at the A5 pulley level (Figure 4). The A1 pulley is partially incised to facilitate the introduction of the catheter into the sheath. The catheter is then advanced until its tip reaches the peripheral incision, at which point irrigation is initiated. Completion of the A1 pulley incision is performed after the irrigation procedure. Release of the tourniquet is essential to confirm adequate digital perfusion. In rare cases, where the skin blood flow of the affected finger is compromised, due to inflammatory edema or as a consequence of irrigation, an additional incision between the two initial ones may be performed. This incision should be limited to the subcutaneous tissue to relieve the pressure.

## Discussion

PFT is a surgical emergency, and despite advances in both antibiotic therapy and surgical management, shifting from open debridement to less invasive closed irrigation techniques, we are still not at the point where substantial morbidity can be reliably avoided [1].

Systematic use of antibiotics may serve as the sole early treatment in mild infections or as an adjunct to debridement and irrigation in more severe cases [6]. 

Surgical treatment of PFT using catheter irrigation via a limited open technique has been reported to result in fewer complications and revisions, shorter hospitalization, faster recovery of hand function, and reduced scar formation compared with open surgery [1,3]. 

However, technical challenges in closed irrigation persist. Numerous irrigation aids have been described, many of which are specialized or not universally available. We identified several such adjuvants in our targeted literature review of previously described irrigation aids, and we provide a table that is meant to serve as a concise reference (Table 1). 

**Table 1:** Reported instruments and modifications described to facilitate closed irrigation of the flexor tendon sheath. This table is intended as a concise reference summary of available technical aids and adaptations reported in prior studies. It does not represent a comparative evaluation of effectiveness, safety, or clinical outcomes between the listed tools.

No.	Year	Author [Ref]	Modifications - Adjuvants
1	2006	Agarwal [7]	Flexor sheath irrigation collection system
2	2006	Mullett [8]	Stainless steel wire
3	2012	Hussain [9]	Wishbone introducer
4	2014	Jagodzinski [10]	Buchanan cholangiogram cannula
5	2014	Warbrick-Smith [11]	Baron obturator stylet
6	2014	Chung [12]	Catheter fenestration
7	2015	Jing [13]	Metal ear suction catheter
8	2019	Haines [4]	Lacrimal probe
9	2020	Knight [14]	Neurosurgical angled cannula
10	2025	Kreutz-Rodrigues [15]	Facial fat grafting cannula

**Figure 5:**
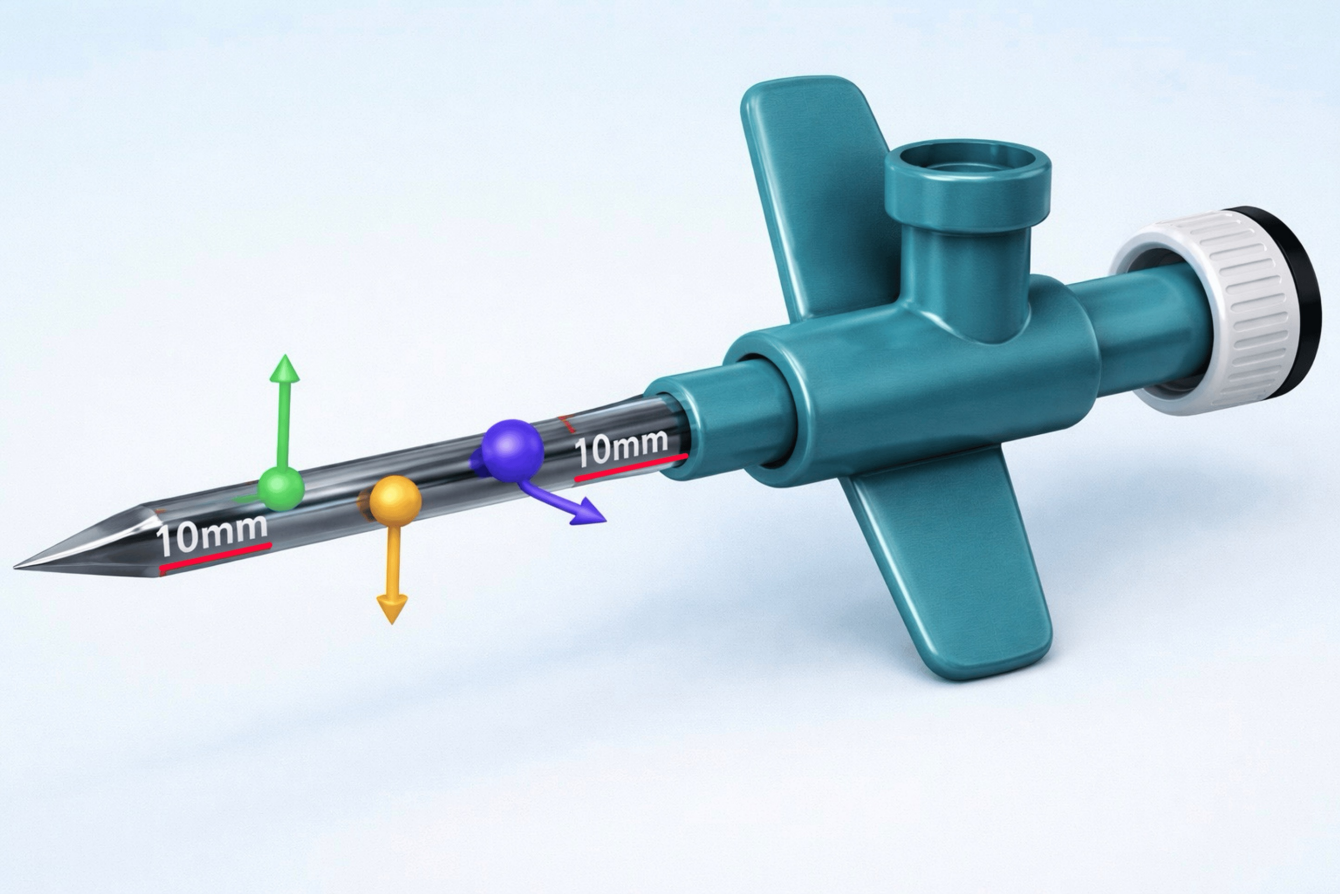
Illustrative depiction of the proposed modified angiocatheter for closed irrigation in pyogenic flexor tenosynovitis. The modified angiocatheter demonstrates multidirectional fluid outflow (different hole colors), designed to potentially enhance the distribution of irrigation solution within the flexor tendon sheath. Red lines depict the areas where we propose that no holes are created. Original image created by the authors for this study.

Angiocatheters are frequently used in closed irrigation of PFT. Here, we present a cost-effective and widely available solution in the operating room, which is intended to theoretically distribute irrigation across multiple exit points (Figure 5).

The modification should be considered an unvalidated procedural option and not a proven enhancement of standard catheter irrigation. Potential limitations include unknown structural integrity after hole drilling, unpredictable flow characteristics, and a lack of mechanical testing. The authors acknowledge that modifying a commercially manufactured device constitutes an off-label intraoperative adaptation, which is sometimes necessary in surgical practice but lacks formal regulatory endorsement.

## Conclusions

Management strategies for PFT continue to vary considerably.

Early initiation of treatment is of paramount importance. Antibiotic treatment, initiated empirically and later tailored to culture results, is now an integral component of all treatment protocols. Variation lies primarily in the timing of surgery and the choice between open debridement and closed irrigation.

Despite advances in conservative and surgical management, complication rates remain substantial, and a universally accepted treatment strategy has yet to be defined.

Further research is required to better define the role of antibiotics as a standalone therapy in selected cases.

A clear, evidence-based algorithm to guide operative management, including procedural selection and timing of intervention, has yet to be established.

Closed irrigation remains an important component of surgical management for PFT. This report describes a simple and feasible angiocatheter modification; however, the technique has not been formally validated, and any potential advantages remain theoretical.

Mechanical testing, fluid-dynamic analysis, and clinical evaluation are necessary before any conclusions regarding the safety or effectiveness of the proposed angiocatheter modification can be drawn.
